# The Influence of Academic Emotions on Learning Effects: A Systematic Review

**DOI:** 10.3390/ijerph18189678

**Published:** 2021-09-14

**Authors:** Jing Tan, Jie Mao, Yizhang Jiang, Ming Gao

**Affiliations:** 1 College of Sports Engineering and Information Technology, Wuhan Sport University, Wuhan 430079, China; 2020410165@whsu.edu.cn; 2 School of Artificial Intelligence and Computer Science, Jiangnan University, Wuxi 214122, China; yzjiang@jiangnan.edu.cn; 3 College of Sports Science and Technology, Wuhan Sports University, Wuhan 430205, China; Gaoming418@whsu.edu.cn

**Keywords:** academic emotions, learning effects, facial expressions, cognitive, systematic review

## Abstract

Academic emotions can have different influences on learning effects, but these have not been systematically studied. In this paper, we objectively evaluate the influence of various academic emotions on learning effects and studied the relationship between positive and negative academic emotions and learning effects by using five electronic databases, including WOS, EMBASE, PubMed, PsycINFO, and Google Scholar. According to established standards, a total of 14 articles from 506 articles were included in the analysis. We divided the 14 studies into nine intervention studies and five observational studies; five of the nine intervention studies found that students who used active learning materials performed better and had higher mental loads than those who used neutral learning materials. Positive academic emotions promoted the learning effect. Four of the five observational studies with high school, college, and postgraduate participants reported that regulating academic emotions can improve learning effects. In conclusion, this paper holds that positive academic emotions are better than negative academic emotions at improving academic performance. In future research, a new method combining multichannel video observation, physiological data, and facial expression data is proposed to capture learners’ learning behavior in various learning environments.

## 1. Introduction

### 1.1. Academic Emotions

The psychologist Reinhard Pekrun believes that academic emotions are all kinds of academic emotional experiences that students feel in learning or teaching situations [1]. When considering academic emotions, we should not ignore the two dimensions of academic emotions: arousal and valence. Academic emotional valence refers to whether the stimulus is pleasant or unpleasant, while academic emotional arousal describes the academic emotional intensity that a stimulus can cause [2]. Based on this classification, emotions can be divided into four groups [3]: positive arousal emotions (e.g., enjoyment, pride), positive emotions (e.g., relaxation), passive arousal emotions (e.g., anger, anxiety), and negative emotions (e.g., extreme depression, despair).

In educational settings, academic emotions are considered to be a key factor affecting learning [1], and a lot of studies have shown that positive academic moods experienced by learners are capable of promoting learning [1,3]. Studies on the role of multimedia learning have shown that inducing positive academic emotions can accelerate learning [4,5,6].

### 1.2. Learning Effects

If the purpose of education is to accelerate students’ learning, then the goal of testing is to assess students’ learning results. Learning effects refers to individual changes in knowledge, skills, emotional attitude, or values after learning the knowledge points [7]. The evaluation of the learning effect can be based on scores in testing, and the skill class is mainly to provide learners with operational training for scoring.

Many studies have shown that cognition, including multimedia learning [4,6,8,9], problem solving [10], influence [11], N-UNCOUNT [12], estimation and policy-making ability [13,14,15], cognitive flexibility [16], complex learning [17], and memory [18,19], play important roles in academic emotion in many aspects. Studies have shown that potency (from passive to active) and arousal (from quiet to excited) have different results in the cognitive process, and different effects on the relevant neural matrix [20,21,22,23]. Some researchers have emphasized the role of arousal and strengthening emotional memory [24,25,26]. These other factors may play a significant role in memory and cognition [27].

Yerkes and Dodson [28] put forward the inverse U hypothesis that there is a negative quadratic relationship between arousal and learning. Moreover, in terms of the optimal arousal level, an easier learning mode has a higher arousal level than a more difficult learning mode. Different intervention conditions can induce a positive arousal effect, thus improving learning performance. A lot of related research results show that there is a strong relationship between academic emotions and learning effects, but causality has not been widely studied, and whether academic emotions are suppressed or promoted for learning success has always been an elusive question [2]. Nevertheless, some studies have not taken negative academic emotions into account [4,6,8]. Negative academic emotions [29] usually lead to poorer attention to learning materials [30], distraction [31], and reduced efficiency, leading to poor learning performance. In addition, Zeng et al. [32] argued that high-intensity academic emotional load unrelated to learning tasks can impair learning performance. Passive and positive academic emotions have negative effects on consciousness [33] and memory [34]. Studies have shown that negative academic moods can stimulate learners to improve learning effects by adjusting their learning strategies [35,36,37]. Furthermore, studies have clearly stated that negative academic moods have no significant impact on learning effects [38]. Therefore, we believe that negative academic emotions can have various effects on learning. Positive academic moods generally have beneficial effects on the cognitive process and studying [4,6,8,11,12]. For instance, Park et al. [8] found in an eye movement study that positive academic emotions before learning can produce a much better learning effect in exams, and the retention of textual information is longer.

Recently, it has been suggested that motivation and academic emotions are linked, because motivational behaviors involve negative and positive academic emotions [39]. Specifically, positive academic emotions may be profitable in most cases, but negative academic emotions, such as dissatisfaction and uneasiness, can have contradictory effects. Moreover, it should be noted that students’ performance often responds to their academic emotions. For example, success can increase expectations of future success, which in turn increases hope and reduces uneasiness. Positive academic emotions may be important for learning and achievement, and academic emotions other than uneasiness may be of equal importance even in areas of negative impact. They may be important for both cognitive and motivational components of self-control learning. Specifically, positive academic emotions may be important for developing intrinsic and sustained motivations. Therefore, in order to better understand how different academic emotions affect learning effects, we included studies of negative and positive academic emotions to analyze the roles played by various academic emotional states in the learning process.

### 1.3. Facial Expressions

For the capture of academic emotions, both subjective and objective methods can be used. Facial expressions are a powerful nonverbal communication method and provide a lot of information about subjective personal experience (e.g., mental state, interest, viewpoint, physiological state, emotions). The development of artificial intelligence technology adds credibility to the recognition of various human emotions through facial expressions [40], indicating the feasibility of using objective methods to capture academic emotions. However, studies of academic mood have long-term dependence on self-reporting [41,42]. Self-reporting is a valuable data source, but the desire to analyze students’ academic emotions in real time during the learning process has created a demand for other forms of academic emotional analysis. As a result, facial expressions, often seen as emotional derivatives, have become a viable channel for such exploration. Since Ekman put forward the general concept of using facial expressions as a data source, people have become more and more interested in facial expressions, and with the development of facial recognition technology over the last 10 years, there has been some related research in the field of education [43,44,45,46]. Especially in scientific experiments, we find that when the experimental results are revealed, the dominant expression of students is first surprised, then negative, and the probability of knowledge change is higher [47]. Surprise, sadness, and disgust are also key facial expressions used to predict the change in students’ knowledge based on conflicting scenarios [48].

It is worth noting that previous studies tended to compare post learning performance with pre learning ability and investigated the impact of academic emotions on learning; they rarely considered the dynamic learning process. Therefore, this study asks whether the influence of academic emotions is stable throughout the whole learning process. In addition, with different levels of learning tasks, the regulating effect of academic emotions is also different. Despite the increasing popularity of this research topic, there is no way to systematically evaluate the different effects of positive and negative academic emotions in different learning stages. Given that the impact of academic emotions on the benefits of learning effects is unclear, we conducted a systematic review of current interventions with control trials and observational studies.

## 2. Materials and Methods

The system evaluation is based on the preferred reporting items of system evaluation [49] and meta-analysis (PRISMA). What is noteworthy is that there is considerable heterogeneity in terms of the research design and participant characteristics, as shown below. Therefore, in this systematic review we did not carry out a complete meta-analysis, but only a test of the main effect size of the intervention studies to supplement the results.

### 2.1. Literature Searches

In order to obtain high-quality research, conventional statistical methods of literature informatics were used. Since there was no target literature in the Chinese database that met the standard, the English database was searched. In this study, “facial expression” and “learning effect” were used as the keywords. First, the retrieval was carried out in WOS, with the formula as follows: TS = ((“facial expression” OR “emotion”) AND (“Learning Outcome*” OR “Learning Effectiveness*” OR “Learning Achievement*” OR “Learning Effect*” OR “Learning Performance*” OR “Learning Gain*”)). Time span: 1900–2020. Then a supplementary retrieval was conducted in the EMBASE, PubMed, PsycINFO, and Google Scholar databases. All of the documents were imported into Endnote document management software to conduct textual research, and duplicate or obviously unrelated documents were screened out. Authors J.T. and J.M. reviewed abstracts from each of the remaining studies. When there were differences, the authors discussed them until reaching a consensus. Then, we used the same method to search all the articles collected by the author of the relevant references, in order to find further relevant articles and avoid omissions.

### 2.2. Article Selection Criteria

First, the two authors J.T. and J.M. narrowed down the number of articles according to our criteria: (a) Intervention (both experimental group and control group) and observational experimental studies (review articles and theoretical articles excluded), published in full in an English-language peer-reviewed journal; (b) related to facial expressions, academic emotions, teaching, and learning (literature related to purely algorithmic facial expression recognition excluded); (c) reports on indicators that reflect learning effects and achievement, such as academic performance or other quantitative indicators, which must be analyzed simultaneously. The literature was screened according to the selection criteria, and especially for this new topic, we included all relevant articles in order to have a comprehensive and profound understanding of it. The systematic review did not limit participants by age, gender, race/ethnicity, etc. When the two authors reached a consensus, the studies were eventually included. The two authors (J.T. and J.M.) extracted information such as year of publication, location, characteristics of participants, learning materials, environmental attributes, intervention conditions, facial expressions, academic emotions (assessment tools), and study results for the subsequent analysis.

### 2.3. Methodological Quality

The two authors J.T. and J.M. independently evaluated the quality of the method according to the criteria of the two scoring tools. For any differences in the quality scores, the authors discussed them to solve the problem. The two authors independently assessed the methodological quality of the intervention study using the PEDRO scale [50]. The Pedro scale has 11 items, namely, randomization, covert distribution, baseline equality, instructor blindness, participant blindness, outcome evaluator blindness, 85% retention rate, intention to treat analysis, intergroup comparison, point measurement, and variability measurement. The score is up to 11 points (detailed description = 1; undetailed description = 0) [50]. The CASP checklist was used to assess the methodological quality of observational studies [51]. It includes three parts (a total of 12 items): the reliability of the research results, what the research results are, and the applicability of the research results. The scoring tables for the evaluation studies are shown in Table 1 and Table 2, with a maximum score of 12 points.

## 3. Results

### 3.1. Study Selection

**Figure 1:**
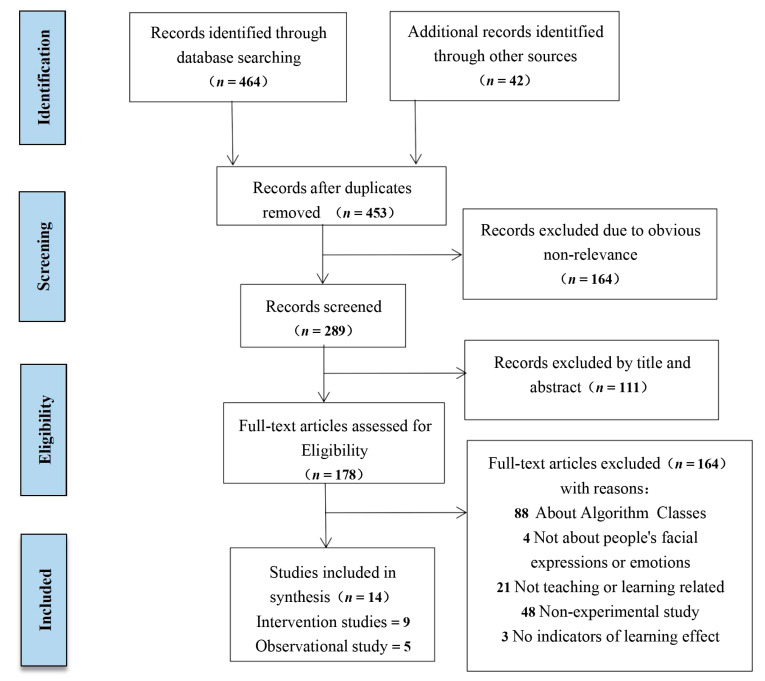
Research screening flowchart.

A flowchart of the research screening process is shown in Figure 1. The method first retrieved 464 possible studies from the WOS, and then found another 42 related studies by searching other databases and consulting references to the most relevant studies. After deleting duplicate and unrelated studies, 289 articles remained. Of these, 178 were considered potentially relevant. Two reviewers (J.T. and J.M.) independently evaluated the 178 articles according to previously established inclusion criteria and excluded 164 articles; the remaining 14 articles met the systematic evaluation. The 14 studies included nine intervention studies [2,52,53,54,55,56,57,58,59] and five observational studies [35,36,37,38,60] According to the predetermined inclusion criteria, two researchers independently completed the study screening. In order to resolve differences in the research, the third author issued a statement and reached an agreement.

### 3.2. Characteristics of Included Articles

As mentioned above, nine of the 14 studies were intervention studies [2,52,53,54,55,56,57,58,59]. The nine intervention studies included three studies involving middle school students [52,53,54], five involving college students [2,55,56,57,58], and one involving graduate students [59]. In two studies involving high school students and three involving design college students [2,53,54,55,58], the intervention program was conditioned on inducing learners’ academic emotions and behaviors by giving them learning materials. Four other studies [52,56,57,59] were conducted in different learning environments to monitor and intervene in academic emotions. Of the five observational studies [35,36,37,38,60], one involved elementary school students [60] and four involved high school and college students [35,36,37,38].

Among the 14 studies, 11 were published after 2015. From facial expressions combined with academic emotions [40], we began to consider the individual characteristics of learners. A total of 1760 participants were enrolled in the 14 studies. The sample size of the study participants ranged from 20 to 458, and the participants ranged in age between 12 and 33.5 years old. In the 14 studies, a total of 13 related indicators were used to measure all aspects of cognition in the learning process: academic mood, strategy, motivation, achievement, attention, evaluation, efficacy, accuracy, reaction time, mental load and effort, learning effectiveness, learning memory, and cognitive load. The characteristics of these interventions and observational studies are summarized in Table 1 and Table 2.

According to the Pedro scale, the mean methodological quality score of the nine intervention studies [2,52,53,54,55,56,57,58,59] was 7.1 on a scale of 4–9. The score is shown in Table 1. According to this CASP listing, there are 12 evaluation tools [35,36,37,38,60]. The five observational studies [51] had an average methodological quality score of 8.2, ranging from 6 to 11. The score is shown in Table 2.

### 3.3. Research Findings

#### 3.3.1. Intervention Research

##### Main Effect Test

This review examined the main effects of nine intervention studies in order to examine the effectiveness of learners’ academic emotions in promoting learning effects (Table 3). In terms of learning performance, the average effect size of learners’ academic emotions was 0.55. Both the upper and lower limits of 95%CI were greater than 0, indicating that the effect size was not caused by accidental factors. The *p*-value of double-tailed test is less than 0.0001, indicating that the promotion effect of adding intervention condition on learning performance is significantly higher than that of no intervention. In terms of academic emotion, the average effect size of learners’ academic emotion was 0.14. The upper and lower limits of 95%CI were also greater than 0, indicating that the effect size was not caused by accidental factors. The *p*-value of the double-tailed test is 0.03, indicating that, after adding the intervention condition, learners also received significantly higher academic emotional regulation in the learning process than without the intervention condition.

##### Heterogeneity Test

Heterogeneity tests were conducted for the two outcome variables (Table 4). The Q test was found to be significant, indicating significant heterogeneity of effect sizes. For learning performance, the I^2^ value is 85, indicating that the variation caused by a real difference in effect size accounts for 85% of the total variation in learning performance. For academic emotion, the I^2^ value is 69, indicating that the variation caused by the real difference of effect size in the regulation of academic emotion in the learning process accounts for 69% of the total variation. Given the boundaries of low, medium, and high heterogeneity [61], the proportion of true difference of effect size in the two types of outcome variables in this study belongs to medium or high, which further indicates a high degree of heterogeneity. Therefore, it is reasonable to adopt the random effects model in this study.

Of the nine intervention studies using subjective methods (academic emotions scale, self-reporting method) to measure learners’ academic emotions, five (60%) [2,53,54,55,58] showed that academic learning materials that represent emotional valence (positive, negative, and neutral) influence students in a lot of ways, with better effects than when using active learning materials with neutral students—the mental load value was higher. However, in one study [53], the use of active learning materials (positive visual design) had a significant influence on shift, while in another study [54], there was no significant influence on shift. Three of the nine intervention studies [52,53,54] were conducted on middle school students in an online learning environment (e.g., web pages, games, and programs), and the results consistently showed that students improved their learning effects in terms of intervention conditions that promoted their own positive academic emotions. Five intervention studies involved college students [2,55,56,57,58], which showed that positive academic emotional state had a promoting effect on performance. One study involving graduate students [59] reported that adjusting the academic emotional valence improved their learning effects. Three positive activation studies [2,55,58] found that there was a positive correlation between mood and performance. From a practical point of view, the design of the active learning materials in the worst case is harmless, but in the best case it can improve the learning effect. Influencing the learning efficiency is not a matter of the learning material itself, but the evolution of learners’ academic emotional state.

In addition, in one study [57], students were given a corresponding stimulus (such as hearing the heart speed up, static, or fake feedback) to academic emotions to check the biofeedback influence on learning performance. The researchers found that false feedback can have a positive impact on learning performance. Biofeedback can promote the current academic mood and performance of learners. One of the studies [56] found that, in an environment where positive academic emotions induced learners’ positive academic emotions, the test scores of the experimental group were higher than those of the control group: their performance in the learning process was higher and their effort was lower. Another study found that, based on online and traditional classes and difficult learning materials [59], the improvement of learning effect was related to the active and flexible use of learning strategies after academic emotion regulation. The methods of improving learning strategies in a state of frustration and anxiety were more significant in the traditional education group. Higher anxiety levels predicted more meaningful strategy use but were not significant in the online education group. There were significant differences in the path coefficient from extrinsic utility value to anxiety between the two groups. The path coefficient was not significant and negative in the online education group, while it was significant and positive in the traditional education group. The learners’ academic emotions played a key role in improving the learning of difficult subjects. Finally, an intervention study on the grouping of learners’ sensitivity to likes and dislikes was conducted in college students [58], with the results showing that positive and negative academic emotions have distinct effects on new word learning. Only in the shallow categories of new word learning tasks does the induced positive academic emotion have a significant tendency to improve the learning effect. On the contrary, induced negative academic emotions generally have an overall inhibitory effect on the learning of new words.

Of the nine interventional studies, two [52,57] found that adding individualized environmental attributes according to learners’ characteristics could more effectively improve learning effects (i.e., learning performance and learning memory). In Cheng et al. [52], students who learned through games performed significantly better than those in the control group in the delay test, and their performance in the learning process was better than that in the previous study. Meanwhile, reasoning texts [57] demonstrated through empirical evidence that learners’ performance was superior to that of the control group. In the context of reasoning problems and text problems, learners were given physiological feedback (such as heartbeat and academic mood), which had a positive impact on learning effects. There were no significant differences in terms of gender, academic emotion, and cognition between the four groups. In addition, in traditional practical courses, some studies [56] proved that push–pull mobile learning courses were developed with a blended learning plan, and diversified learning channels were provided for students through a push–pull mobile learning system. During the learning process of the application of this project, there was a high frequency of positive academic emotional achievement (such as happiness, satisfaction, self-confidence, and optimism). “Group positive academic emotional performance” was significantly and highly positively correlated with “knowledge achievement” and “experimental work achievement.” The learning effect of students in the experimental group was significantly better than that of the control group.

#### 3.3.2. Observational Research

Of the five observational studies, three [35,38,60] used subjective methods (the self-reporting of academic emotion scale) to measure learners’ academic emotions. One study [36] used objective methods (facial expression recognition software) to assess learners’ academic emotions. The final study [37] combined subjective and objective methods to measure learners’ academic emotions. Two studies (20%) [35,37] found that learners expressed more negatively activating academic emotions (such as anger and anxiety) and passively passivating academic emotions (such as sadness, frustration, and depression) during the learning process. Instead of actively activating academic emotions (such as happiness), anxiety has a significantly positive effect on learning; learners with angry expressions have the highest learning effect, and passively activating academic emotions can improve the learning effect. One study [36] showed that, after positive events (positive action results) occurred in the learning process, compared with negative events, learners’ confused academic emotions were significantly different, but had no effect on happiness or depression. The higher the degree of confusion after positive action, the higher their performance would be. However, other studies have found that students have different academic emotional experiences according to different tasks of learning materials, although it is not necessarily guaranteed that the reciprocal academic emotional valence states will change immediately after active induction and regulation [60]. In the face of favorite learning materials, learners will have higher cognitive focus and concentration [38], and academic emotional valence will change under self-regulation, but it is not so closely related to performance. These related indicators affect students’ learning effect. Different learning environments and materials will lead to different effects of academic emotions on learning results. In a game-based learning environment, learners’ situational academic emotions (such as confusion, anxiety, and anger) affect learning effects and improve learning performance after positive behavioral outcomes are made [36,37]. In addition, another study [35] found that frequent use of learning materials with positive academic emotions would directly affect learners’ performance, while negative academic emotions (such as anxiety) would improve learners’ comprehension scores.

Three of the five observational studies on high school and college students reported that adjusting the academic emotional valence can improve their learning effects [35,36,37]. These studies measured academic achievement emotions, game scores, before and after test scores, duration, engagement, and motivation. Only one observational study was conducted on primary school participants [60], to explore how the negative task-related emotional experience of students before cooperation increased the group’s academic emotional regulation in cooperation, and negative group interaction negatively affected the students’ academic emotional experience after the task. The results showed that there was a significant negative correlation between task-related academic emotional experience and the percentage of moderated negative socio-academic emotional interaction before the task, while there was no significant correlation between post-task academic emotional experience and the percentage of moderated negative socio-academic emotional interaction. However, one study [38] found that learning materials that combine learners’ preferences affect learning strategies and performance but are not closely associated with performance outcomes. In the learning process, there are many aspects (e.g., behavior, participation, and cognitive focus) of performance that can be different.

## 4. Discussion

This study systematically estimated the influence of the interaction of academic emotions on participants’ learning strategies, learning motivation, self-efficacy, achievement, and other aspects on learning effects in the learning process. This study reviewed 14 studies and found that eight out of nine (90%) interventional studies and one (10%) observational study supported positive academic emotions in promoting learning effects. Two intervention and observational studies [38,54] suggested that learners’ academic emotions had no significant influence on learning effects. In addition, Taub et al. [36] and Ahn and Harley [37] used facial expression recognition software to study, while Marchand and Gutierrez [59] and Saito et al. [35] found that negatively activating academic emotions (e.g., confusion, anxiety, and anger) had a positive and significant effect on learning effects, but no significant effect on positively activating academic emotions (e.g., happiness) and passively passivating academic emotions (e.g., sadness and depression). Compared with negative academic emotions, although existing evidence tends to support the notion that positive academic emotions may have more of an effect on certain aspects of learning effects, because of environmental and intervention conditions, and how learning materials and measuring tools are used to assess learning effects in specific areas, it is too early to come to any firm conclusions. In general, the findings of this systematic review indicate that learners’ academic emotions may differ due to different periods of study, different learning materials, and different learning environments, and thus have different influences on learning effects.

Only one observational study [60] has compared the academic emotions of primary school students in group learning. In addition, two interventional studies [52,53] compared the influence of learners’ academic emotions on learning effects for junior high school students. Except for one observational study [53], both interventions agreed that, under the intervention of different learning materials and learning environments, the learning effect is significantly improved [52,53]. Previous studies have shown that, compared with other aspects of academic emotions, adaptive assisted learning systems tailored to learners’ characteristics have a more significant beneficial impact on students’ learning effects [62,63]. Most of the experiments involved high school and college students, and five of six intervention studies and three of four observational studies showed that academic emotional regulation improved learning effects. Three observational studies [35,36,37] used relevant facial expression recognition software to further study the influence of negatively activated academic emotions (such as anxiety, anger, and confusion) on learning results. Students with angry academic emotions had a statistically higher learning gain ratio than those with sad academic emotions. Research [64,65] showed that, in some cases, academic emotions like anger have the potential to improve learning. These academic emotions affect learning strategies, lead to real learning, and then affect the learning effects. The results show that the academic emotions can cause learning gain. However, during game-based learning, Sabourin and Lester used self-reporting methods to assess students’ academic emotions. The students reported the highest levels of attention and curiosity, followed by confusion, frustration, excitement, boredom, and finally, anxiety. In addition, confusion and boredom were found to be negatively correlated with learning gains [66].

Cognitive imbalance is an uncomfortable state [67], which can lead to negative academic emotions. Cognitive imbalance is a necessary condition for students to deeply understand learning [68]. Negative academic emotions (such as anxiety and disappointment) play an important role in the learning process. Other recent research has examined the effects of confusion and frustration on learning. Research performance, confusion, and frustration bring about better learning effects for learners [69,70,71]. However, this may only apply to certain activities. For instance, if students are confused when reading multimedia materials because they do not understand the content, they may not be able to resolve their confusion, which will cause them to feel frustrated, and finally, bored. This illustrates the importance of analyzing academic emotions in each situation, so that we can understand when learners are confused or frustrated, thus helping students to learn effectively. The difference in academic emotions in the study [36] depends on the positive or negative outcomes associated with solving the puzzle.

However, two other studies [38,54] have shown that academic emotional guidance has no significant influence on learning effects. An intervention study [38] suggested that academic emotional guidance is related to behavior, learning strategies, and cognitive regulation, while an observational study [54] suggested that, after academic emotional guidance occurs, it can improve memory and mental load without affecting learning effects. The evidence in this review extends the knowledge gained from the previous literature and suggests that positive academic emotions may have an advantage over negative academic emotions in terms of learning effects. Frequent participation in active academic emotional activities may influence behaviors conducive to learning strategy regulation [72]. Another intervention study [59] used graduate students as subjects to improve performance by promoting the active and flexible use of learning strategies. In the process of learning, with the increase in the difficulty of learning materials, there will be more negative academic emotions, but students can adopt more adaptive coping strategies to defuse negative academic emotions.

Due to the learning environment being constructed by different intervention conditions (learning forms and learning materials), feature-based academic emotions include the tendency of individuals to make stable and consistent responses in a specific way. In addition, immediate academic emotional response constitutes state-based academic emotion [73]. Cues in learning situations can affect this response and may fluctuate over time. However, feature-based and state-based academic emotions are often very similar, and need to be carefully distinguished [73]. Finally, the research [52] is on state-based academic emotions, focusing on students’ short-term academic emotional experience in a specific game situation rather than their general experience. The academic emotions guided by learning materials focus on what is experienced by students under the particular intervention conditions. However, in certain cases, individual characteristics may be more advantageous. Games have promoted students’ science achievement in the long term [52]. Some studies have proposed a spiral emotional learning model, which includes a right-to-left academic emotional axis level positive price, which explains the correlation between academic emotions and scientific learning [74].

The model consisted of a right-to-left horizontal academic emotional axis of positive valence, a top-to-bottom representational constructive learning, and a bottom-to-top learning vertical learning axis, and identifies four quadrants. At the origin, the third axis of knowledge is perpendicular to these two axes. Some studies have proposed that in the process of science learning, learners’ academic emotions will change with the learning process, and knowledge will be acquired with their movement in the quadrant and spiral up along the knowledge axis [74]. In addition, available evidence suggests that the difficulty of learning materials may have different effects on the use of learning strategies and academic emotional state [58,59]. Due to the challenge of learning materials, it is difficult for students to self-regulate their learning. Therefore, in the game-based learning environment, students can autonomously learn and practice, which enables them to maintain a high level of motivation and participation [75]. However, there is still some debate about which academic emotions may be more effective at improving learning effects. Therefore, from now on, we should make further efforts to clarify the differences and relationships between the two types of academic emotions. In addition, in subsequent studies, the characteristics of academic emotions may need to be considered as a covariable to control their effects.

In summary, the results of this systematic review indicate that, compared with negative academic emotions, positive academic emotions may be more effective at improving certain aspects of learning effects, especially in high school and college students. The research results not only contribute to an understanding of the different learning effects of academic emotions, but also have some practical significance. There is growing support for incorporating the regulation of students’ academic emotions into classroom teaching plans, perhaps through different learning materials, learning environments, teaching methods, and facial recognition tools, to study students’ academic emotions throughout the learning process as an effective way to improve learning effects [52,53,60]. Gee [76] argues that learning should not be divorced from experience, because it is always best when people identify and generalize patterns in a given environment through concrete experience over a long period of time. It is important to note that passive academic emotions may be more conducive to long-term learning and memory, and the beneficial effect on learning effects should not be ignored, even though it may lead to poor academic performance. Therefore, a series of academic emotions (positive and negative) will appear in science learning experience, which may contribute to the learning effect. In addition, the lack of a social component (person–person interaction) in our learning scenarios may lead to more academic emotions being expressed through facial expressions [77]. Social interactions that occur during the learning process may have further positive effects on learning effects [56]. The review also found that few studies currently involved elementary and graduate subjects, so future studies may consider these two groups as potential participants.

## 5. Strengths and Limitations

Although we have outlined the results of research on the relevant aspects of learning effects for both types of academic emotions, the conclusions in this review must be considered in the context of their limitations. First, 5 of the 14 studies (36%) were cross-sectional in design, and 9 of the 14 intervention studies (64%) were identified. However, three out of five (60%) of the included studies showed that positive academic emotions improved some aspect of learning effects, indicating that guiding students to positive academic emotions has great value in further research. Of course, in the above discussion, negative academic emotions should not be ignored. Secondly, among these studies, randomized controlled trials (RCTs) accounted for more than half, publication bias was large, various methods of measuring results were common, and the learning range of learners was large. Therefore, we did not conduct a complete meta-analysis, but only conducted a main effect test.

This review includes interventional studies and observational studies. To maximize interstudy comparison, we focused on the effects of different learning environments that produced positive and negative academic emotions under different intervention conditions on aspects related to learning effects. The lower frequency of academic emotions observed in studies using facial recognition software tools may be because participants’ expressions do not adequately represent the actual incidence of academic emotions experienced, since facial expressions are only one of the expressive components of academic emotions. Faces not showing academic emotions, in fact, do not rule out the inner or physical emotional education experience, especially because it is easier to suppress low-intensity academic emotions, so in a future study, we suggest the use of both subjective and objective measurement methods to measure the learner’s academic emotions, combining self-reporting and physiological measurements (such as facial expression and EEG). In this way, the academic emotional state can be better measured.

## 6. Conclusions

Based on existing interventions and observational studies, this paper systematically evaluates the effects of positive and negative academic emotions on learning effects. This review tends to support the view that positive academic emotions are superior to negative academic emotions in some aspects of improving learning effects. Given that most existing studies use only the self-reporting method to measure academic emotions, we suggest combining subjective and objective methods to achieve more accurate results. From a methodology perspective, this is a novel method to capture learners’ learning behavior in various learning environments by combining multichannel video observation, physiological data, and facial expression data, which are subjective and objective means. In addition, there are few studies on negative academic emotions. The specific circumstances under which negative academic emotions can improve learning effects are unknown, and more rigorous randomized controlled trials and long-term follow-up are needed to further confirm the current findings.

Future research should explore strategies teachers can use to enhance academic emotions and the knowledge of learners and ask (1) how teaching support can be related to students’ academic emotions and (2) how learning from former stages can inform teaching strategies to guide the development of learning behavior. At the same time, learning analysis technology can also be used to understand the behavior of learners in the learning process, so as to contribute to better education and teaching results. Future research should use multimodal approaches to determine how each learner’s motivation and academic emotions change over time and how an increase in academic emotion and motivation interacts with the learning environment. These adaptive environments can also ensure that students engage in effective learning strategies and avoid maladaptive behaviors. In addition, it would be worthwhile to extend the research by looking at the temporal aspects of academic emotions—specifically, using a combination of sensors that capture facial expressions and self-assessment to study how and when academic emotions change, depending on what factors. Observing sequences of academic emotional experiences may further reveal the role of academic emotions in learning.

## Figures and Tables

**Table 1:** Traits of interventional research.

Research (Authors, Publication Year, Methodological Quality, Location)	*N*	Experimental Group 1	Experimental Group 2	Control Group	Intervention Conditions	Facial Expression (Emotional Variable) (1) Tools (2) Mood	Relevant Indicators	Results
		(1) Population (2) Age (3) LS (4) EA	(1) Population (2) Age (3) LS (4) EA	(1) Population (2) Age (3) LS (4) EA				
Junior school (10–15 years old)								
Cheng et al. 2019 [52] 9/11 China (Taiwan)	112	(1) 54 (2) Seventh grade (3) Humunology (4) Web games	None	(1) 58 (2) Seventh grade (3) Words; Pictures; Videos (4) Web page	Does the teacher explain the relevant concepts? A game-based learning environment	(1) Emotion questionnaire (2) Positive/Negative Positive activation of emotions; Positive inactivation of emotions; Negative activation of emotions; Negative inactivation	Emotions; Before the test; After measuring 1; Back 2; Delay testing; Long-term learning memory; Learning effect	The experimental group was superior to the control group in the first and last two experiments. Students who experience positive academic emotions have better learning effects. The learning effect decreases over time.
Shangguan et al. 2020 [53] 7/11 China	223	(1) 29M/50 (2) 13.90 ± 0.68 (3) Flash (4) Programs	(1) 79M/173 (2) 14.75 ± 0.75 (3) Flash (4) Programs	None	(1) VPBP, *n* = 45 (2) VPBN, *n* = 43 (3) VNBP, *n* = 45 (4) VNBN, *n* = 40	(1) Positive Emotion Self-Report Scale (2) Positive enjoyment; Excited; Satisfied; Active; Interested; Relaxed	Emotions; Motivation; Cognitive load; Learning performance	Positive academic emotion can improve the learning effect and promote the motivation and cognitive load.
High school (15–18 years old)								
Beege et al. 2018 [54] 9/11 Germany	162	(1) NA (2) 16.49 ± 0.96 (3) Educational video (4) Learners’ emotions: Positive; Negative	(1) NA (2) 16.49 ± 0.96 (3) Educational video (4) Emotional load of educational videos: Positive; Negative	None	(1) Positive guidance and positive video (*n* = 32) (2) Positive guidance and neutral video (*n* = 51) (3) Neutral guidance and positive video (*n* = 40) (4) Neutral guidance and neutral video (*n* = 39)	(1) Self-assessment model (SAM) (2) Unlucky; Lucky	Emotions; Mental load (ML); Mental effort (ME); Learning effect	Academic emotions do not affect the learning effect, but promote the transfer, memory, and mental load in the learning process.
College (20–32 years old)								
Munchow and Bannert, 2019 [55] 8/11 Germany	145	(1) 65 (2) 20.20 ± 2.60 (3) Multimedia (4) Positive affective induction	(1) 80 (2) 20.20 ± 2.60 (3) Multimedia (4) Neutral affective induction	None	(1) PA (2) CG	(1) PANAS; SEK-27 scale (2) Positive; Negative	Emotional state; Prior knowledge; Emotional regulation; Learning effect	Adjusting learners’ emotions and positive academic emotions can improve the learning effect.
Chung et al. 2019 [56] 5/11 China (Taiwan)	115	(1) 56 (2) College students (3) Ship energy-saving work results (4) Have a positive fuel-saving attitude	None	(1) 59 (2) College students (3) Ship energy saving work results (4) no Positive qualities	Push–pull mobile learning system developed by hybrid learning scheme; Diversified learning channels	(1) Positive Emotions Questionnaire (2) Positive Happy; Content; Confident; Optimistic	Emotions; Learning motivation; Achievement; Learning effect	The oil-saving accomplishment of the experimental group was better than that of the control group. Students in the experimental group had the highest frequency of confidence in positive emotional achievement.
Guo et al. 2018 [2] 9/11 China	20	(1) NA (2) College students (3) Korean words (4) Image system (Learning stages 1, 2)	(1) NA (2) College students (3) Korean words (4) Image system (Learning stages 3, 4)	None	4 different learning stages, alternating emotional image blocks	(1) Positive and Negative Emotion Scale (PANAS) (2) Positive; Neutral; Negative	Emotions; Accuracy; Reaction time	Negative emotional state leads to a decline in academic performance. With the passage of time, the learning effect is very significant, the correctness of learning will increase, and the real-time strategy will decrease.
Strain et al. 2013 [57] 6/11 US	38	(1) 15M/38 (2) 21.80 ± 5.07 (3) Multimedia (4) Text-based problem	(1) 15M/38 (2) 21.80 ± 5.07 (3) Multimedia (4) Inference-based problem	(1) 15M/38 (2) 21.80 ± 5.07 (3) Multimedia (4) Controls respectively,	Whether there is stimulation of auditory heartbeat, static, false feedback	(1) Mood scale (2) Positive Arousal dimension: low arousal/sleepiness -high arousal/activity; Valence dimension: unpleasant-pleasant	Emotion, Metacognition, Performance	Erroneous biofeedback is an effective method to manipulate affective state and metacognitive judgment; it has a positive effect on learning performance and can promote metacognitive judgment.
Silva et al. 2012 [58] 7/11 France	63	(1) 50 (2) 21.50 ± 3.47 (3) Vocabulary (4) Low-sensitivity group	(1) 50 (2) 21.50 ± 3.47 (3) Vocabulary (4) Highly sensitive group	None	Random appearance of neutral words; Aversive words; False words	(1) Emotional scale (2) Positive; Negative Arousal: Low--High	Emotions; Mean reaction time (RTS); Error rate (ERs)	The high-sensitivity group had an inhibitory effect on emotional value, while the negative emotional state had an overall inhibitory effect on new word learning.
Graduate students (22–68 years old)								
Marchand and Gutierrez, 2012 [59] 5/11 France	185	(1) 72 (2) 33.50 ± 9.97 (3) Introduction to research methods (4) Online	(1) 219 (2) 33.50 ± 9.97 (3) Introduction to research methods (4) Traditional	None	Different learning environments	(1) Achievement Emotion Questionnaire (AEQ) (2) Positive; Negative Hope; Anxiety; Anger; Frustration	Emotions; Self-efficacy; Learning strategy; Learning motivation	Negative academic emotions can improve learners’ performance, and anxiety can improve learning results. The influence of negative academic emotions on the use of learning strategies leads to the improvement of academic performance.

**Table 2:** Traits of observational research.

Research (Authors, Publication Year, Methodological Quality, Location)	*N*	Experimental Group 1	Experimental Group 2	Control Group	Experimental Period	Facial Expression (Emotional Variable) (1) Tools (2) Mood	Relevant Indicators	Results
		(1) Population (2) Age (3) LS (4) EA	(1)Population (2) Age (3) LS (4) EA	(1) Population (2) Age (3) LS (4) EA				
Primary school students (12 years old)								
Manty et al. 2020 [60] 8/12 Finland	37	(1) 37 (2) Grade six (3) Collaborative physics task (4) None	None	None	None	(1) Mood scale (2) Positive; Negative; Mixed; Neutral	Social emotional interaction; Emotion regulation	Negative emotional experience increased the emotional regulation in group cooperation, and negative interaction affected students’ emotional experience after the task. The emotional valence does not change immediately.
High school; College (15–26 years old)								
Taub et al. 2019 [36] 11/12 Canada	61	(1) 61 (2) 20.00 ± 1.50 (3) Microbiology (4) Game	None	None	69.40 ± 21.70 (min)	(1) FACET (2) Happy; Frustrated; Confused	Emotions; Emotional duration; Game score; Back; Operation results	The feelings experienced after positive action are different from those experienced after negative action. After the occurrence of negative events, confusion is obviously different, but it does not affect happiness or depression. The degree of confusion after active action can accurately predict the total score of the game.
Ahn and Harley, 2020 [37] 8/12 France	33	(1) NA (2) 20.00 ± 1.64 (3) History apps (4) Multimedia Applications	None	(1) NA (2) 20.00 ± 1.64 (3) Crystal island (4) Game	30 min	(1) FaceReader 7; Self-report questionnaire (2) Sadness; Happiness; Anger; Anxiety; Fear	Facial expressions; Dominant emotions; AOIS; Note; Learning gain	Negative academic emotions can improve the learning effect, and angry learners have the highest learning effect.
Saito et al. 2018 [35] 6/12 Britain	108	(1) 108 (2) High school (3) English (4) Answer the questions in each stage	None	None	Three semesters	(1) Mood scale (2) Happiness; Anxiety	Emotions; Motivation	Strong internalized motivations evoke a variety of emotions (anxiety, happiness). More frequent use of materials with positive emotions has a direct impact on acquisition, and anxiety can improve learning effects.
Ben-Eliyahu and Linnenbrink-Garcia, 2015 [38] 8/12 US	458	(1) NA (2) High school; College (3) Hobby classes (4) Written; Online surveys	None	None	None	(1) Mood scale (2) Positive; negative	Cognitive regulation; Emotional regulation; Cognitive focus; Achievement; Re-evaluate	Higher levels of self-regulation; Students used “reassessment” more often than their least favorite subject; Emotions, behaviors, and cognitive regulation are related to learning strategies, but not closely related to academic performance.

Notes: LS: learning stuff; EA: environmental attribute; NA: invalid; VPBP: Visual positive and behavioral positive emotion design conditions; VPBN: Visual positive and behavioral neutral emotion design conditions; VNBP: Visual neutral and behavioral positive emotion design conditions; VNBN: Visually neutral and emotional design conditions; AOIS: Define Scope of Interest.

**Table 3:** Main effects test in intervention studies (random effects model).

Outcome Variable	*k*	*N*	*p*	*d*	95%CI
learning performance	81	5708	<0.00001	0.55	[0.40,0.69]
academic emotion	54	3654	0.03	0.14	[0.01,0.27]

Notes: *k* represents the number of independent effect sizes, *N* represents the sample size (the same below), *p* represents the double-tailed test, and *d* = Cohen’s D. Here, 95% CI refers to the 95% confidence interval (including lower and upper limit) of effect size *d* corresponding to each outcome variable.

**Table 4:** Heterogeneity test of effect size.

Outcome Variable	Tests for Heterogeneity				
	*Q*	*df* (*Q*)	*p*	*I^2^*	*Tau^2^*
Learning performance	524.68	80	<0.00001	85	0.36
Academic emotion	172.78	53	<0.00001	69	0.15

## Data Availability

Data is contained within the article. For detailed information of each part, please contact the corresponding author.
